# Chemical-sensitive graphene modulator with a memory effect for internet-of-things applications

**DOI:** 10.1038/micronano.2016.18

**Published:** 2016-05-09

**Authors:** Haiyu Huang, Li Tao, Fei Liu, Li Ji, Ye Hu, Mark Ming-Cheng Cheng, Pai-Yen Chen, Deji Akinwande

**Affiliations:** 1 Department of Electrical and Computer Engineering, University of Texas at Austin, Austin, TX 78712, USA; 2 Maxim Integrated Inc., Dallas, TX 75240, USA; 3 School of Medicine, Stanford University, Stanford, CA 94305, USA; 4 Houston Methodist Hospital Research Institute, Houston, TX 77030, USA; 5 Department of Electrical and Computer Engineering, Wayne State University, Detroit, MI 48202, USA

**Keywords:** chemical sensing microsystems, CVD graphene, graphene field-effect sensors, internet of nano-things, microsensor networks, RF and analog microdevices

## Abstract

Modern internet of things (IoTs) and ubiquitous sensor networks could potentially take advantage of chemically sensitive nanomaterials and nanostructures. However, their heterogeneous integration with other electronic modules on a networked sensor node, such as silicon-based modulators and memories, is inherently challenging because of compatibility and integration issues. Here we report a novel paradigm for sensing modulators: a graphene field-effect transistor device that directly modulates a radio frequency (RF) electrical carrier signal when exposed to chemical agents, with a memory effect in its electrochemical history. We demonstrated the concept and implementation of this graphene-based sensing modulator through a frequency-modulation (FM) experiment conducted in a modulation cycle consisting of alternating phases of air exposure and ethanol or water treatment. In addition, we observed an analog memory effect in terms of the charge neutrality point of the graphene, *V*_cnp_, which strongly influences the FM results, and developed a calibration method using electrochemical gate-voltage pulse sequences. This graphene-based multifunctional device shows great potential for use in a simple, low-cost, and ultracompact nanomaterial-based nodal architecture to enable continuous, real-time event-based monitoring in pervasive healthcare IoTs, ubiquitous security systems, and other chemical/molecular/gas monitoring applications.

## Introduction

Advanced nanostructures such as two-dimensional carbon nanomaterials, namely, graphene^1^, with their single-molecule-level sensitivity in chemical sensors and biosensors^2–7^, may establish a foundation for future ubiquitous micro-/nanosensor networks^8,9^. In general, the sensor, modulator, and memory that are necessary components of a sensor network are separate and independent modules^10,11^. For example, a graphene chemical sensor would need to be combined with a silicon-based modulator to make it compatible with wireless radio frequency (RF) operation for data synchronization in internet-of-things (IoTs). However, in the case of nanosensors^2–6^ based on nanomaterials and nanostructures, their small sizes and heterogeneous features compared with conventional solid-state electronic devices could make their integration particularly challenging^12,13^. To integrate nanomaterial sensors with commercial silicon-based modulation interfaces, complicated post-transfer processes must be performed^13^; this results in a high-cost and non-compatible nodal structure and therefore limits the applicability of nanomaterial sensors for the internet of nano-things (IoNT)^14^.

It has recently been reported that graphene field-effect transistors (GFETs) can exhibit not only ultrahigh mobility but also interesting gapless electron-hole spectra and ambipolar transport properties^1,3,6^, which lead to a nonlinear effect in the control of the gate voltage with respect to the drain current. On the basis of this unique property, special modulation functions, such as full-wave rectification, frequency mixing, amplitude modulation (AM), frequency modulation (FM), and phase modulation (PM), can be directly achieved with a single graphene device, which is not possible for conventional semiconductor devices^15–19^. Here we propose a different modulation mechanism utilizing chemical gating, instead of the application of an electrical signal, to directly modulate the input carrier signal by means of the chemical dopants (for example, gases or molecular substances) to which a GFET is exposed. This monolithic all-graphene paradigm can enable virtually simultaneous *in situ* chemical sensing and signal modulation, thereby significantly reducing the integration complexity and cost, which is of great interest for ubiquitous sensor network applications.

For most graphene sensor applications, the sensor output signal, in information theory, is one-dimensional (1D), in terms of either direct current (DC) or electrical resistance. This 1D spectrum offers limited information processing, storing, and distributing capabilities^2–7,20–23^ because of the low-signal dimensionality. One advantage of the proposed chemically modulated sensing device, compared with other nanomaterial sensors, resides in its two-dimensional (2D) output signal, which provides information through both the amplitude and frequency of an RF signal. Very recently, Lee *et al.* have demonstrated that electrically gated modulation on graphene exhibits a transition from a fundamental input tone to a second-harmonic output tone^19^, which enables AM to FM functions using a single device. Here we show that this signal transition is not abrupt and that the fundamental and second-harmonic tones instead coexist, with gradually varying strengths depending on the potential level of the graphene, which can be modulated by means of a chemical (or electrical) gating effect. In this scenario, mixed AM/FM modulation can be achieved and the amplitude responses at both frequencies constitute a 2D output signal, thereby improving the overall reliability and the tolerance to environmental noise and interference.

We also investigate for the first time the memory effect of chemically gated graphene devices, in which the shift of the charge neutrality point of the graphene (*V*_cnp_) depends not only on the chemical agents to which it is exposed but also on the exposure time, in an integral fashion not found in other semiconductor devices. This interesting feature could be advantageous for continuous-time biomedical and environmental monitoring, considering that the calibration of the memory effect on site is regarded as an important issue for practical applications in long-period and multicycle sensing. Thus far, several methods of tuning *V*_cnp_, on which the modulated output signal depends, have been reported, including annealing the device under high vacuum, post-heating at high temperature, and applying a specialized gas/wet treatment after the adsorption of substances onto the graphene^1,3,24,25^. However, these calibration methods require extreme conditions to initiate the memory effect in the graphene and thus are not feasible for pervasive sensing or continuous monitoring applications. A simple and robust approach that can be used to precisely calibrate the graphene device would be preferable.

**Figure 1:**
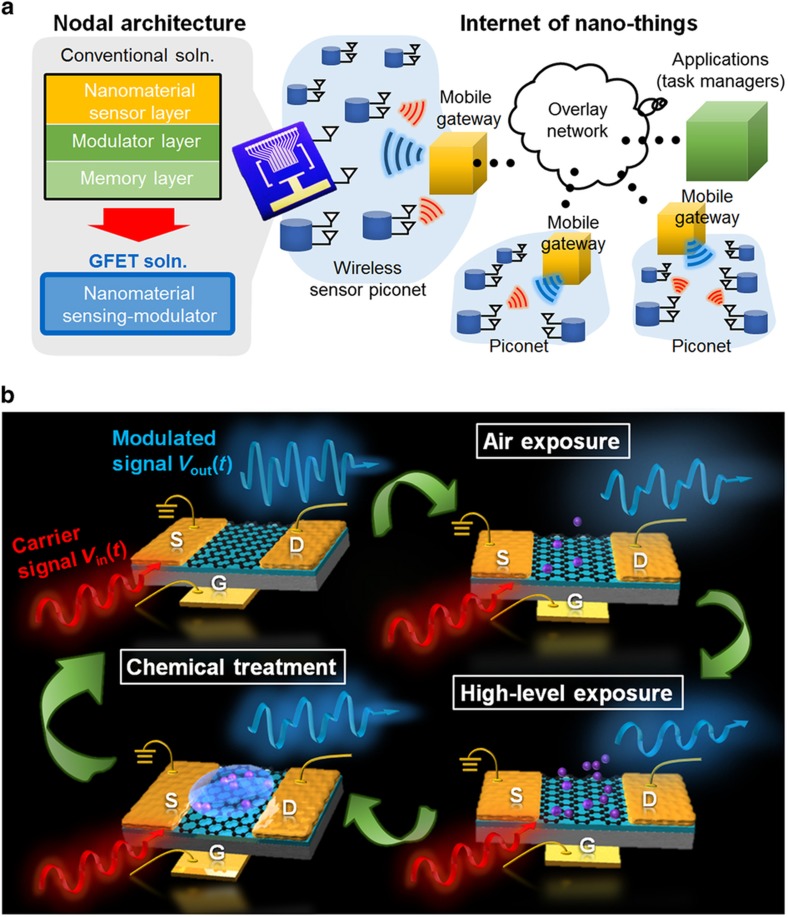
System overview: (**a**) The nodal architecture of an Internet of Nano Things consisting of nanomaterial sensing modulators. (**b**) Conceptual illustration of the chemical gating modulation of a single graphene device, in which a high-frequency input carrier signal is applied at the back gate without any DC gate bias and the modulated output at the drain varies throughout a modulation cycle consisting of alternating phases of gas exposure and wet chemical treatment.

Here, we introduce and demonstrate a chemical-gating-enabled self-modulation concept, which simultaneously offers sensing, modulation, and memory functions within a single device, permitting at least one or two stages of a sensor node module to be eliminated. Unlike traditional nanosensors that generate a static current output (which requires at least one stage of a signal-converting module for wireless reception), this graphene-based sensor no longer requires an FM modulator because the sensor output is already an RF signal (second-harmonic tone), which is fully compatible with wireless communication media^26^. Moreover, with the application of a gate-voltage pulse sequence as an effective electrochemical means of controlling the charge transfer and capacitive gating of the graphene, the memory effect of the proposed device can be precisely calibrated under air exposure or wet treatment. This eliminates the need for costly temperature- or vacuum-controlled equipment, as mentioned above. The mixed sensing/modulation of this device via chemical gating, along with the precise electrochemical calibration of the graphene, shows promising potential for application in IoNTs for ubiquitous sensing in the healthcare, automobile, security, and food preservation industries, among others. Figure 1a shows the architecture of a practical network consisting of a large number of sensor nodes. From the system perspective, the proposed graphene-based sensing modulator may greatly simplify the nodal architecture for IoNTs, as depicted in Figure 1a, thus enabling enormous savings in cost, integration effort, and power consumption.

## Materials and methods

### Mixed modulation concept based on the chemical gating of graphene

Figure 1b illustrates the basic operation principle of the proposed device: the carrier signal is a single-tone sinusoidal (RF) wave applied at the back gate of a GFET, which is compatible with wireless or high-speed wireline communication systems. Under a given drain bias, the output drain current waveform consists of both fundamental and second-harmonic frequency components that are modulated by the chemical gating. The adsorption of p-type substances (for example, oxidizer-type gas molecules) will cause a gradual decrease in the amplitude of both tones at the output, with the second-harmonic tone being the first to be fully suppressed. By contrast, the adsorption of n-type substances (for example, electron-donor-type chemical agents) will reverse the modulation of the output, restoring it to its original condition. These two phases essentially form a complete chemical modulation cycle, in which the two different phases, depending on the type of application, can be utilized for sensing and for the resetting (neutralization) of the sensor. For instance, in the sensing of oxidizing materials, materials with abundant electron donors may be used to reset the sensor. Similarly, in the sensing of electron-donating materials, an oxidizing material may serve as the reset agent.

**Figure 2:**
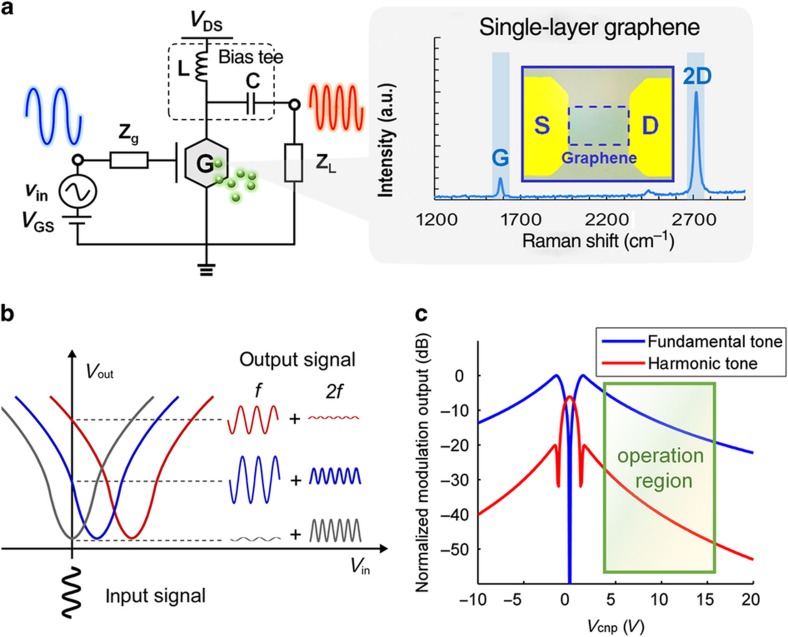
Principle of mixed modulation via the chemical gating of graphene. (**a**) The circuit diagram; the inset shows a microscopic image of a GFET and the Raman spectrum of the CVD graphene channel. (**b**) Mechanism of mixed AM/FM GFET modulation; gray: if *V*_cnp_ is zero, then the output consists only of a strong harmonic tone; blue: when *V*_cnp_ shifts away from 0 V, a large fundamental tone is present and the harmonic tone decreases; red: when *V*_cnp_ shifts higher, both the fundamental tone and the harmonic tone decrease, but the harmonic tone decreases more rapidly. (**c**) Physics-based modeling result (assuming electron-hole symmetry) for the two-dimensional modulation output (in terms of a normalized output voltage) depending on the *V*_cnp_ shift; a region is outlined in which both the fundamental and harmonic tones monotonically decrease, which can be used as the operation region for a demonstration of chemical modulation.

Figures 2a and b, respectively, show the circuit diagram for this graphene sensor and how the ambipolar transport behavior of the GFET, with a ‘V-shaped’ drain output–gate voltage characteristic curve, may lead to frequency doubling. From Figure 2b, we note that the magnitude (modulation level) of the output signal at the doubled frequency is controlled by chemical gating, which involves a shift in *V*_cnp_. When a single-tone input at a frequency *f* is applied to the gate with zero DC gate bias, the modulated output shows a visible change as *V*_cnp_ is altered through chemical gating: (1) if *V*_cnp_ is zero, then the drain output has a vanishing component at *f* and a strong component at 2*f* (gray curve); (2) if *V*_cnp_ is up-shifted above the critical point, then the signal component at *f* is amplified, whereas the 2*f* tone decreases (blue curve); (3) if *V*_cnp_ is far from the critical point, then the output signal begins to monotonically decrease, exhibiting drops at both *f* and 2*f*. With a large shift in *V*_cnp_, the second-harmonic tone is completely suppressed, as the fundamental tone continues to decrease (red curve).

A theoretical analysis of the mixed modulation output as a function of *V*_cnp_ is shown in Figure 2c; the results were obtained using a compact, physics-based GFET model^27^. The best-signal linearity is observed at two critical conditions: the peaks in the fundamental-tone curve (blue shaded region in Figure 2c) and the dips in the harmonic-tone curve (red shaded region in Figure 2c; *V*_cnp_=±1.4 V). This variation in nonlinearity is explained by the schematic diagram presented in Supplementary Figure S1a. The *V*_cnp_ of graphene can be shifted from positive to negative values, over a wide range, by means of exposure to p-/n-type dopants of different concentrations. As a result, there exists an ideal operating region in which both components are monotonic functions of *V*_cnp_, as shown in the gray shaded region in Figure 2c.

In addition to the chemical-gating-induced *V*_cnp_ shift, the variation in carrier mobility also affects the output RF signal of the GFET. In fact, although the attachment of most chemical agents to graphene can affect its *V*_cnp_^14^, there is no material that shows a measurable impact on graphene’s carrier mobility^15^. The results calculated for the two-tone output via 2D vector mapping (Supplementary Figure S1b) clearly distinguish the traces of the *V*_cnp_ shifts at different mobility values. Our theoretical results indicate that the corresponding *V*_cnp_ can be retrieved from the 2D output even in the presence of variations in other parameters (for example, the carrier mobility), thereby demonstrating excellent robustness in sensing compared with conventional approaches involving the detection of only 1D outputs (for example, DC currents).

### Device fabrication

For our experiment, the GFETs were fabricated using a shadow mask without any lithography step to preserve the pristine surface of the graphene. This is because in addition to the H_2_O/O_2_ redox system, residues such as photoresist could also contribute to the formation of charge traps. The synthesized graphene was first transferred, using polymethyl methacrylate as a support, onto a SiO_2_/Si substrate with a 285-nm thermal oxide layer as the dielectric to form a back-gate device structure. Raman spectroscopy with a 442-nm blue laser was used to confirm the quality of the graphene after transfer (see the inset of Figure 2a), revealing a G peak at 1582 cm^−1^ and a symmetric 2D band at 2695 cm^−1^ (bandwidth of ~32). Back-gated field-effect transistors were formed by means of the electron-beam evaporation of 2-nm Ti+48-nm Au metal contacts using a shadow mask to measure the electrical properties of the devices. The area of the metal contacts was 200 μm×200 μm, which defined the graphene channel width; the devices were fabricated with various channel lengths of 25, 50, 100 and 200 μm. Further details regarding the graphene preparation^28–30^ and device fabrication can be found in our previous work^31^. The intrinsic sensitivity of a graphene chemical sensor can be extremely high^6^. However, in this study, our focus was on demonstrating a novel multifunctional sensor/modulator module and its applicability for the sensing of time-varying chemical events rather than demonstrating the high intrinsic sensitivity of graphene-based devices.

### Measurement setup

In the chemical-gating-enabled self-modulation demonstration, the GFET was vacuum fixed on a probe station and the back-gate and drain contacts of the GFET, which had a channel length of 100 μm, were connected to bias tees to combine and separate the RF and DC signals. The input RF signal was generated by a function generator, and the DC drain bias was generated by a voltage source. The output signal at the drain was measured using an Agilent DSO-X 3034A digital oscilloscope (Agilent Technologies, Santa Clara, CA, USA) with the spectrum analyzing function based on the fast Fourier transform. In the *V*_cnp_ calibration demonstration, the DC drain current to gate voltage (*I*_DS_–*V*_GS_) curves of the GFET were recorded using the HP4145B semiconductor parameter analyzer under ambient conditions, with a drain-to-source bias of 100 mV. Droplets of 200-proof ACS/USP ethyl alcohol (100% ethanol), as the wet treatment agent used in the experiment, were directly applied to the device on the probe station in the ambient environment at room temperature. We note that the test bench setup was suitable for kHz modulation frequencies. However, the GFETs are applicable at higher frequencies, potentially up to several GHz^18^, which would require a different measurement setup. In practice, the frequency of the input/output signals of the modulator will depend on the transmission medium. For example, if the sensor-modulator is used in a low-frequency RFID communication system, then the operating frequencies will typically be in the kHz range. However, if it is used in a Bluetooth system, then the corresponding frequencies will be 2.4 GHz/4.8 GHz.

## Results and discussion

### Air exposure-wet treatment chemical gating on a GFET

**Figure 3:**
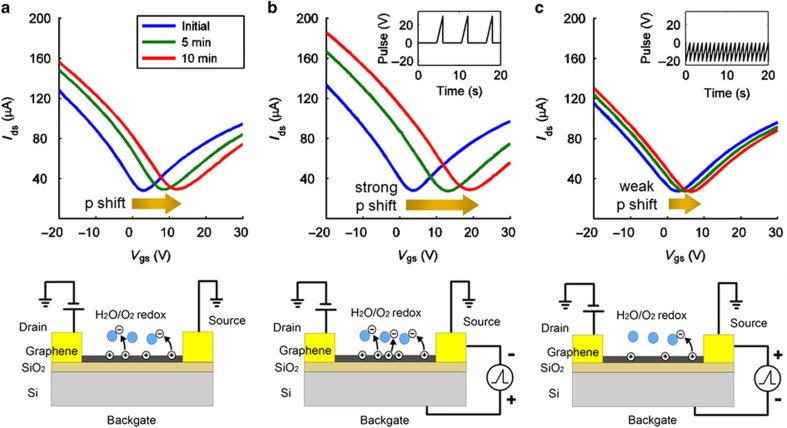
Demonstration of the pulse-sequence-adjustable electrochemical memory effect in a GFET during 10 min of air exposure. (**a**) The H_2_O/O_2_ redox system adsorbed from the air serves to form charge traps that attract electrons transferred from the graphene, resulting in a p-type shift of *V*_cnp_. (**b**) With the application of a positive-gate-voltage pulse sequence, electrons are temporarily pumped into the graphene channel from the source metal contact and the electric field on the graphene surface points toward the air region, accelerating the electron charge transfer from the graphene to the H_2_O/O_2_ charge traps during a positive-voltage pulse. Because a large population of electrons has been transferred to charge traps, more holes are accumulated on the graphene layer when the pulse ends, resulting in a stronger p-type shift of *V*_cnp_ than in the case without a positive-voltage pulse. (**c**) With the application of a negative gate-voltage pulse sequence, holes are temporarily pumped into the graphene channel and the electric field points toward the substrate, decelerating the charge transfer from the graphene to the H_2_O/O_2_ charge traps and resulting in a weaker p-type shift of *V*_cnp_.

The chemical gating effect typically results from the adsorption of an oxidizer (for example, NO_2_, O_2_, and ambient air) or an electron donor (for example, NH_3_ and NH_2_), inducing charge transfer between the graphene and the adsorbed substance^6–8^. For instance, under ambient air, the *I*_DS_–*V*_GS_ curve of a GFET naturally shifts rightward (p-type shift) over time, as verified by the DC characteristic measurement shown in Figure 3a. The device under investigation was a large-area back-gate GFET^31^ patterned directly with a shadow mask, which exhibited reliable device characteristics (see Supplementary Table S1 and the Supplementary Information). A back-gated device, in which the surface of the single-layer graphene is left uncovered, is particularly suitable for chemical gating tests. The rate of electron transfer from the graphene layer to the H_2_O/O_2_ redox system^32^ sourced from the ambient air is proportional to the overlap between the occupied states of the graphene and the unoccupied state of the H_2_O/O_2_ solution^32–34^. Hence, the rate of the p-type shift of *V*_cnp_ exhibits an exponential decay until the graphene density of states (DOS) and the H_2_O/O_2_ redox-coupled DOS reach equilibrium. In our test, *V*_cnp_ had undergone a marked shift from 3 to 12 V after 10 min of air exposure.

**Figure 4:**
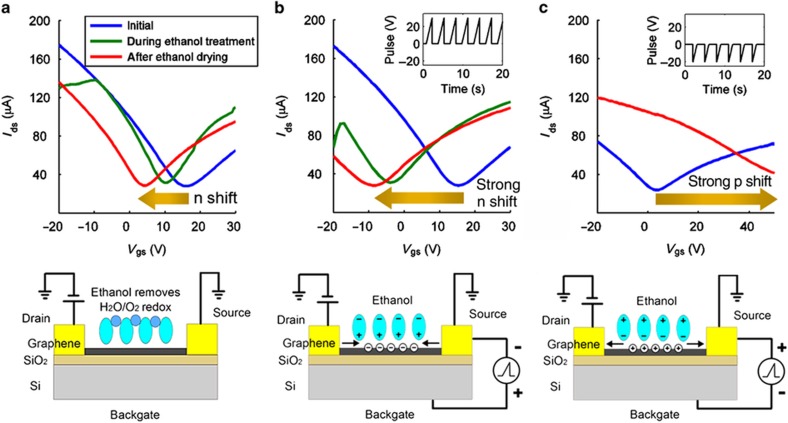
Demonstration of the pulse-sequence-adjustable electrochemical memory effect in a GFET during 20 s of wet ethanol treatment. (**a**) The H_2_O/O_2_ residues adsorbed on the graphene are readily soluble in ethanol, and because the charge traps are removed after the ethanol is dried, the *I*_DS_–V_GS_ curve shows an n-type shift (the recovery of the initially p-doped GFET). (**b**) With the application of a positive gate-voltage pulse sequence, the dipole moments of the ethanol molecules, which point in the direction of the air region, are gradually enhanced and the capacitive electrostatic gating effect draws electrons from the metal contacts to move to and accumulate on the graphene surface. Combined with the effect in (**a**), the overall n-type shift of *V*_cnp_ is larger after ethanol (wet) recovery with positive-voltage pulses. (**c**) With the application of a negative gate-voltage pulse sequence, the dipole moments of the ethanol molecules are reversed, pushing electrons away from the graphene layer. Now, the capacitive gating effect leaves a large number of holes accumulated on the graphene, resulting in a very strong p-type shift of *V*_cnp_.

There are several ways to restore the intrinsic electrical characteristics and tune the *V*_cnp_ of graphene; these include (1) high-vacuum annealing or high-temperature heating, which causes the desorption of the substances on the graphene^1,24^, and (2) wet chemical treatment, although most studies of this method are based on photoresist-contaminated GFETs and the recovery chemicals involved are hazardous (BOE, HF, and CHCl_3_)^25,35^. In this study, ethanol treatment could serve as a cost-effective, non-toxic and ecofriendly method of resetting the *V*_cnp_ of a photoresist-free GFET. During each wet treatment phase, a naturally p-doped GFET was treated with an ethanol droplet for 20 s, followed by a drying process using a nitrogen gun. We found that the instantaneous measured *V*_cnp_ could be reduced from 16 to 3 V, as seen in Figure 4a. This is because the ethanol solution eliminates the H_2_O/O_2_ redox system on the graphene surface and hence shifts the Fermi level of the graphene to its original energy level prior to air exposure, as depicted in Supplementary Figure S2. Unlike conventional BOE treatment^14^, which relies on the chemical reaction between HF and H_2_O^14^, here the H_2_O/O_2_ redox system is removed because it is readily soluble in ethanol; therefore, this wet treatment is a chemical-reaction-free and environmentally friendly process that is suitable for practical applications.

### Realization of a mixed modulation cycle of air exposure and ethanol treatment

**Figure 5:**
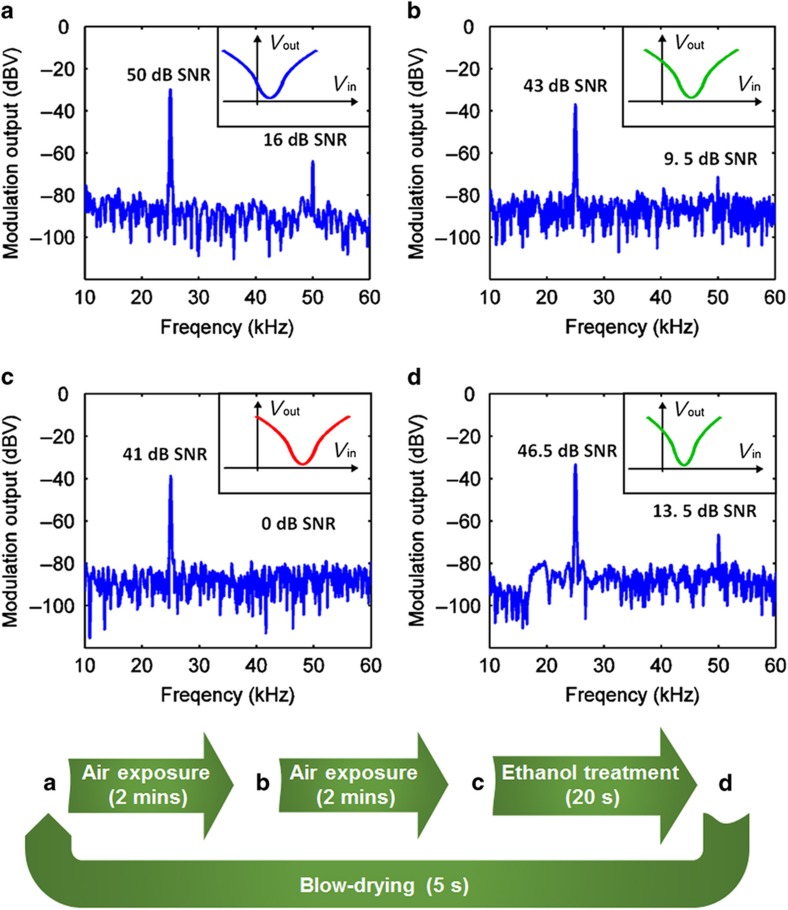
Realization of a mixed AM/FM modulation cycle of alternating phases of air exposure and wet treatment using a chemically gated GFET with a single-tone back-gate input carrier signal at 25 kHz. (**a**) Initially, when *V*_cnp_ is approximately 3 V, the output vector is <50 dB, 16 dB> SNR, with a noise level of −80 dBV. (**b**) *V*_cnp_ shifts to a higher voltage after 2 min of exposure, and the output drops to <43 dB, 9.5 dB> SNR. (**c**) Continued air exposure for a further 2 min results in an even higher *V*_cnp_ and an output of <43 dB, 0 dB> SNR. (**d**) The operation conditions can be reversed by means of ethanol-based wet treatment. At the moment when an ethanol droplet is applied, the intermediate output is <46 dB, 13.5 dB> SNR. After 20 s of treatment, the ethanol is blown dry for 5 s, after which the device is reset to the original state of <50 dB, 16 dB> SNR. SNR, signal to noise ratio.

Here, mixed modulation controlled by chemical gating is demonstrated in an air exposure-ethanol recovery modulation cycle using the original GFET represented in Figure 3a, without loss of generality for other gases and wet chemicals that may also exert similar modulation effects in this GFET. Figure 5 shows the spectra of the modulated output signals for a monotone 25-kHz input signal with a 3-V peak-to-peak voltage at the back gate, under a 100-mV DC drain bias. The initial *V*_cnp_ of the fabricated GFET is ~3 V, and the output vector, consisting of the fundamental tone at 25 kHz and the second-harmonic tone at 50 kHz, has a signal-to-noise ratio (SNR) of <50 dB, 16 dB>. Figures 5a–c represent the air-exposure modulation phase, during which the output vector decreases to <43 dB, 9.5 dB> SNR after 2 min (Figure 5b) and to <41 dB, 0 dB> SNR after a further 2 min (Figure 5c). Figures 5a–c show that surprisingly, the signal strength of the second-harmonic tone, which is a good indicator of the level of charge or chemical exposure, may be detuned from 16 to 0 dB because of the shift in *V*_cnp_. Typically, the shift in *V*_cnp_ is quite sensitive to different chemical events, as will be discussed below. Figures 5c and d represent the ethanol treatment phase, during which the output vector is reset back to <50 dB, 16 dB> SNR after the wet treatment. Figure 5d is the output spectrum obtained in an intermediate state during wet treatment. Finally, after the ethanol is fully dried, the output is reset to its original signal shape (Figure 5a). This modulation cycle was repeated several times, and the spectral responses were nearly identical (Supplementary Figure S3). The 2D output signal <SNR_f1_, SNR_f2_> may provide redundant information for signal post-processing and analysis in sensing systems, which may improve their robustness and enable absolute accuracy in sensing; details of the decoding of sensing signals are provided in the Supplementary Information.

### Pulse-sequence-enabled analog memory effect with *V*_cnp_ as the state indicator

The *V*_cnp_ value of a graphene device can be used as an analog state to label its level of chemical exposure over a finite period of time, having a role similar to that of an analog memory. More interestingly, this memory-like effect is adjustable with the assistance of a voltage pulse sequence applied at the gate. Under ambient air exposure, the charge traps on the graphene surface predominantly consist of the H_2_O/O_2_ redox system. Here we demonstrate that one can dynamically control the hysteresis-like behavior of the GFET by repeatedly applying a unidirectional positive (negative) voltage-pulse sequence, resulting in a faster (slower) *V*_cnp_ shift during air exposure. Figures 3a–c show *V*_cnp_ shifts measured under identical air exposure conditions and time durations but with voltage-pulse sequences of different polarities applied at the back gate. Figure 3a shows the *I*_DS_–*V*_GS_ curve shifts at different points in time during air exposure without the application of gate-voltage pulses. Figure 3b shows that the trend of the *I*_DS_–*V*_GS_ curve shifts with the application of a positive gate-voltage pulse sequence, where each pulse is swept from 0 to 30 V (each cycle consisted of a 1.5-s positive pulse and a 4.5-s idle period, corresponding to a total of 10 pulses per min). Figure 3c shows that the trend of the *I*_DS_–*V*_GS_ curve shifts with the application of a negative gate-voltage pulse sequence, where each pulse is swept from −20 to 0 V (each cycle consists of a 1-s negative pulse and a 0-s idle period, corresponding to a total of 60 pulses per min). When the gate-to-source voltage pulse is positive and the drain-to-source DC bias is small, electrons are temporarily pumped into the graphene channel and the inward electric field (with respect to the air regions) near the graphene layer will preferentially accelerate the electron charge transfer^32,33^ from the graphene to the H_2_O/O_2_ charge traps. When the pulse ends, the accumulated electrons transferred from the graphene to the charge traps cause a stronger p-type shift of *V*_cnp_ compared with that in the case without a gate-voltage pulse (Figure 3a). The readily shiftable nature of *V*_cnp_ results in a highly sensitive chemical gating modulator. By contrast, if the gate-to-source voltage is negative, then holes are temporarily pumped into the graphene channel by a negative pulse and the direction of the electric field on the graphene surface is outward, resulting in a weaker p-type shift during air exposure. As a result, the application of a positive or negative gate-voltage pulse sequence may influence the extent of the p-type shift of *V*_cnp_, which, in turn, affects the modulated RF output of the chemically gated modulator.

The behavior of the graphene in the wet chemical treatment phase can also be adjusted using a gate-voltage pulse sequence. From Figure 4a, we already know that ethanol treatment may be used to recover a p-doped GFET nearly to its intrinsic state. With the application of a positive gate-voltage pulse sequence during ethanol treatment, *V*_cnp_ may even be pushed below its intrinsic value. Figures 4a–c compare the *V*_cnp_ shifts observed under identical wet ethanol treatment conditions but with (Figure 4a) no gate pulses, (Figure 4b) a positive pulse sequence, and (Figure 4c) a negative-pulse sequence. It is clearly seen from Figure 4b that when a positive-voltage pulse sequence is applied during the 20-s ethanol treatment (each cycle consists of a 1.5-s pulse and a 1.5-s idle period, corresponding to a total of 7 pulses during the ethanol treatment), *V*_cnp_ experiences a strong n-type shift (from +16 to −8 V) once the ethanol has dried. This change is marked compared with the unbiased scenario represented in Figure 4a (which shows a recovered *V*_cnp_ of 3 V). This phenomenon has previously been investigated through hysteretic voltage sweeping, which shows that when an external electrical field is applied to a polarized ionic liquid on a graphene surface, the capacitive gating effect can lead to an increase in the carrier density of the graphene^32^. Similarly, in the ethanol treatment applied here, the capacitive (ion) gating enables rapid control of the carrier density through repeated unidirectional voltage pulses. Because ethanol is also a polar solvent, when a positive pulse is applied at the back gate, the inward electric field causes the molecular dipoles in the ethanol droplet to orient toward the graphene surface, thereby increasing the electron carrier density in the graphene channel and consequently enhancing the n-doping effect observed after treatment under a positive pulse sequence, as shown in the inset of Figure 4b. As the number of applied positive pulses increases, the accumulated dipole moment becomes stronger. Thus, in Figure 2b, the redox removal process (identical to that in Figure 2a) is combined with capacitive gating, which results in a stronger n-type shift compared with that seen in Figure 2a. After the ethanol solution has dried, the relaxation of the n-type carriers and the re-accumulation of H_2_O/O_2_ redox charge traps from the ambient air are triggered simultaneously.

By contrast, with the application of a negative gate-voltage pulse sequence during ethanol treatment, the *V*_cnp_ shift is reversed from an n-type shift to a strong p-type shift, with a final *V*_cnp_ higher than 50 V, as shown in Figure 4c. As illustrated in the inset of Figure 4c, under the influence of the outward electric field, the dipoles are oriented in the opposite direction of the graphene surface, repelling free electrons away from the graphene. In this case, a very high density of holes is induced in the graphene channel as a result of the strong p-type doping caused by the negative pulses.

### Toward continuous monitoring applications based on GFET Modulators with a memory effect

**Figure 6:**
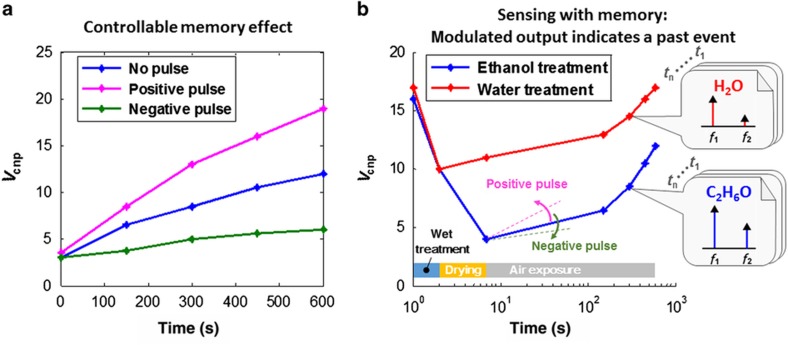
Application example of sparsely sampled monitoring of wet chemical treatment. (**a**) Memory effect controlled by an electrical pulse (gate-voltage) sequence (for example, reset of the delay time) during ambient air exposure. (**b**) A short wet treatment (with ethanol or water) event can be monitored based on the modulation output at any sample point during the air exposure phase. The application potential of the device as a sparsely sampling nanomaterial monitor with a memory effect is clearly demonstrated: any data read during air exposure contains information regarding the previous wet treatment.

The memory effect of GFET modulators is sensitive to both electrical gate bias and chemical gate conditions. The benefit of this feature for IoT-based continuous monitoring applications can be seen from the time-domain presentation of the experimental data shown in Figure 6. Figure 6a presents the time-domain response of *V*_cnp_ during air exposure for the same GFET represented in Figure 3a, with the application of different electrical pulse (gate-voltage) sequences. It is evident that the electrical bias conditions, for example, the pulse sequence polarity and amplitude, can be used to tune the analog memory effect (that is, the time-dependent history of *V*_cnp_) during air exposure. Figure 6b compares the results of the wet treatment-air exposure modulation cycle using different wet treatment solvents: ethanol (C_2_H_6_O) and water (H_2_O). The different capabilities of ethanol and water in removing the H_2_O/O_2_ redox system from the graphene surface may lead to different profiles of the *V*_cnp_ shift over time, resulting in very different event-driven modulation cycles. It is seen from Figure 6b that the results of sampling at time points during air exposure can clearly indicate the previous chemical event (ethanol or water treatment). The GFET modulator, which combines multiple functions of sensing, frequency modulation, and self-memory, may allow operation at much lower power with sparser data sampling (Figure 6b) compared with traditional power-hungry sensor systems, which typically require sampling at a much higher rate for continuous-time monitoring.

The proposed multifunctional graphene module, with its highly simplified integration and operation stages (Figure 1a), arguably suits the demand for low-power pervasive IoNTs. For this single-GFET device, the only persistent power consumption is for the drain-to-source DC bias, which is only on the order of 10 μW (0.1 V×0.1 mA). This low-power feature makes the device compatible for connection with existing communication systems, for example, USB, Bluetooth, and WiFi. Moreover, a fully passive frequency-doubling component can be achieved using a diode-connected GFET or a GFET-based quad-ring mixer, which draws power directly from the RF interrogating source^36^. The passive sensor modulator is very suitable for integration with communication systems containing a wireless interrogating power source, for example, RFID or NFC. Considering the large numbers of sensor nodes required in IoTs or IoNTs, the power savings over a long period of time could be enormous. The use of either an active or passive modulation scheme will depend on the application type and the system-level design for sensing and communication. For example, for typical indoor sensing applications, a lithium-ion battery or any other power source can be conveniently connected to the drain terminal of the GFET to provide DC power. For other power-critical applications, such as state-of-the-art bio-implantable devices, the use of on-site batteries is not possible and a passive GFET modulator is more desirable.

We also considered, as another example, two different protein solutions, namely, milk and ESAT-6 (Ref. 37; a biomarker of *Mycobacterium tuberculosis*), as a third material to be added to the chemical gating modulation cycle. For the sake of brevity, the measurement results are presented in the Supplementary material (Supplementary Figure S4). We found that within 2 h of ambient air exposure, the difference in the *V*_cnp_ of the GFET could provide information regarding to which type of protein the graphene may have been exposed. In this context, this nanomaterial sensor node may have great potential to become part of future point-of-care platforms. Smart phones or other portable gateways could receive the sensor signal and instantly decode and synchronize the information to the cloud, providing a seamless, convenient and ultra-low-cost healthcare IoT solution in the big data era.

## Conclusions

We have demonstrated, for the first time, a multifunctional nanosensor-modulator that combines (RF) frequency modulation, sensing, and an analog memory effect to record the history of various chemical events. This new frequency-modulated sensing paradigm may enable the development of practical graphene-based nanosensors with wireless connectivity for the continuous monitoring of surface chemical events. We have demonstrated in an air exposure-wet treatment cycle that the modulated chemical gating of the proposed graphene field-effect device and the resulting RF output signal may provide reliable diagnoses of different types of treatment options and solvents, providing a promising route toward infection and biometric monitoring^38,39^ in healthcare applications. The low maintenance cost and low energy consumption of this smart graphene sensor, with its advantageous hybridization of sensing/frequency-modulation/memory capabilities, are compatible with the demands for distributed and ubiquitous continuous monitoring. Our demonstration reveals the unique possibility for multifunctional nanosensor-modulators to serve as ultracompact and low-cost nano-nodes for real-time, event-based monitoring in pervasive healthcare IoTs and other chemical/molecular/gas sensing applications. A systematic investigation of a large variety of nanomaterials will be beneficial for future attempts to develop this concept into practical sensing products that offer enhanced specificity in modulated chemical gating.
