# Supplementary figures and images for: Chemical-sensitive graphene modulator with a memory effect for internet-of-things applications
Source: Microsyst Nanoeng. 2016 May 9;2:16018. doi: 10.1038/micronano.2016.18 (PMC6444719; doi:10.1038/micronano.2016.18)

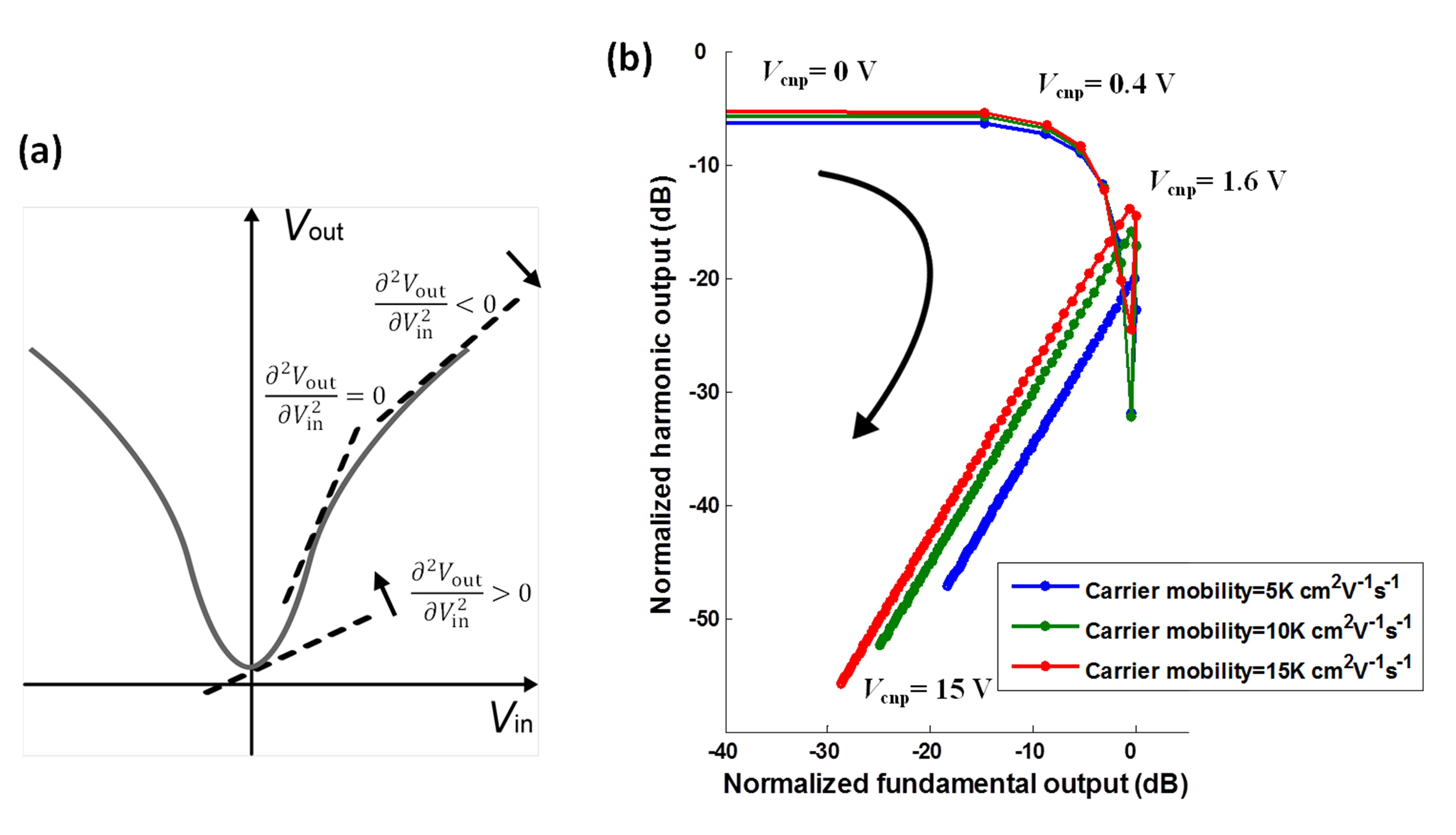

Supplement: Supplementary Information [file micronano201618-s1.tiff]
